# Spontaneous Regression of a Retinal Mass With Epstein-Barr Virus-Positive and Cytomegalovirus-Positive Panuveitis in Methotrexate-Associated Lymphoproliferative Disorder

**DOI:** 10.7759/cureus.103392

**Published:** 2026-02-10

**Authors:** Ryunosuke Watanabe, Takashi Koto, Hiroshi Keino

**Affiliations:** 1 Department of Ophthalmology, Kyorin University School of Medicine, Tokyo, JPN

**Keywords:** cytomegalovirus, epstein-barr virus, extranodal, mtx-lpd, uveitis

## Abstract

Methotrexate (MTX) has been widely used for patients with autoimmune diseases as a corticosteroid-sparing agent. We here report an 86-year-old Japanese woman treated with MTX for rheumatoid arthritis who presented with panuveitis along with a yellowish retinal mass. Both Epstein-Barr virus (EBV) and cytomegalovirus (CMV) were detected in aqueous humor and vitreous fluid by quantitative polymerase chain reaction. The retinal mass gradually regressed over eight months after MTX withdrawal. The present case indicates that intraocular MTX-lymphoproliferative disorder (MTX-LPD) should be considered in the differential diagnosis of patients presenting with uveitis who are undertaking long-term MTX therapy. Multiplex PCR along with quantitative PCR for intraocular fluids may be helpful for understanding the pathogenesis of intraocular MTX-LPD.

## Introduction

Methotrexate (MTX) has been widely used in patients with autoimmune diseases as a corticosteroid-sparing agent, and MTX is a standard therapy for rheumatoid arthritis [1]. MTX-associated lymphoproliferative disorder (MTX-LPD) develops in patients with rheumatoid arthritis treated with MTX [2]. Infection with Epstein-Barr virus (EBV) has been associated with MTX-LPD [3]. Although extranodal lesions are common in patients with MTX-LPD [4], MTX-LPD in intraocular tissues is extremely rare [5]. We report a case of rheumatoid arthritis treated with MTX that showed spontaneous regression of a retinal mass in the eye, with Epstein-Barr virus (EBV)- and cytomegalovirus (CMV)-positive panuveitis, after MTX withdrawal.

## Case presentation

**Figure 1:**
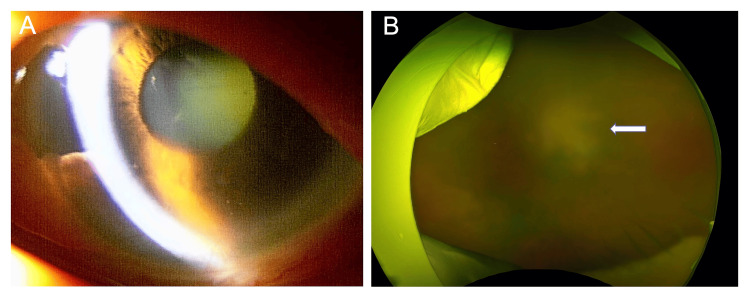
Slit-lamp and fundus images in the left eye at presentation. (A) Slit lamp photograph showing corneal edema, pigmented keratic precipitates on corneal endothelium. (B) Ultra-widefield fundus image of the left eye showing large yellowish  mass (white arrow) in the posterior pole.

An 86-year-old Japanese woman who had been treated with MTX (4 mg/week) for 10 years and abatacept for 5 years for rheumatoid arthritis presented with complaints of decreased vision in the left eye. On initial examination, the best-corrected visual acuity (BCVA) was 1.0 (20/20 Snellen equivalent) in the right eye and hand motion in the left eye, respectively. Intraocular pressure was 10 and 28 mmHg in the right eye and left eye, respectively. Slit-lamp examination revealed corneal edema, pigmented keratic precipitates, anterior chamber cells (2+), and cataract with nuclear sclerosis of Grade 4 in the left eye (Figure 1A). Fundus examination showed vitreous opacity with a yellowish retinal lesion in the posterior pole (Figure 1B).

The right eye showed normal ocular findings with an intraocular lens in place. As shown in Table 1, laboratory analysis revealed minor elevations in liver function tests and C-reactive protein (CRP), although other systemic markers, including erythrocyte sedimentation rate, showed no significant abnormalities. While the Toxoplasma IgG test was positive, all other infection screening markers remained unremarkable.

**Table 1:** The laboratory analysis at presentation. WBC: white blood cells, RBC: red blood cells, Hb: hemoglobin, Plt: platelets, ESR: erythrocyte sedimentation rate, BUN: blood urea nitrogen, Cre: creatinine, T-Bil: total bilirubin, AST: aspartate aminotransferase, ALT: alanine aminotransferase, LDH: lactate dehydrogenase, ACE: angiotensin-converting enzyme, CRP: C-reactive protein, STS: serologic tests for syphilis, TP: Treponema pallidum.

Variables	Normal range	Unit	Data
WBC	3300–8600	/μL	4600
RBC	386–492	x 10^4^/μL	343
Hb	11.6–14.8	g/dL	11.3
Plt	15.8–34.8	x 10^4^/μL	14.3
ESR	3–15	mm	8
BUN	8–20	mg/dL	12.8
Cre	0.46–0.79	mg/dL	0.59
T-Bil	0.4–1.5	mg/dL	0.5
AST	13–30	IU/L	42
ALT	7–23	IU/L	30
LDH	124–222	IU/L	273
ACE	7–25	U/L	21.3
soluble IL-2 receptor	141–394	U/mL	370
CRP	0–0.14	mg/dL	0.24
Na	138–145	mEq/L	139
K	3.6–4.8	mEq/L	3.9
Cl	101–108	mEq/L	105
Ca	8.8–10.1	mg/dL	8.9
Mg	1.9–25	mg/dL	1.9
STS			(-)
TP			(-)
Beta-D-glucan	0–11	pg/mL	<6
Toxoplasma IgM	0–0.7	IU/mL	<0.5
Toxoplasma IgG	0–2	IU/mL	3.3
Interferon gamma release assay			(-)

**Figure 2:**
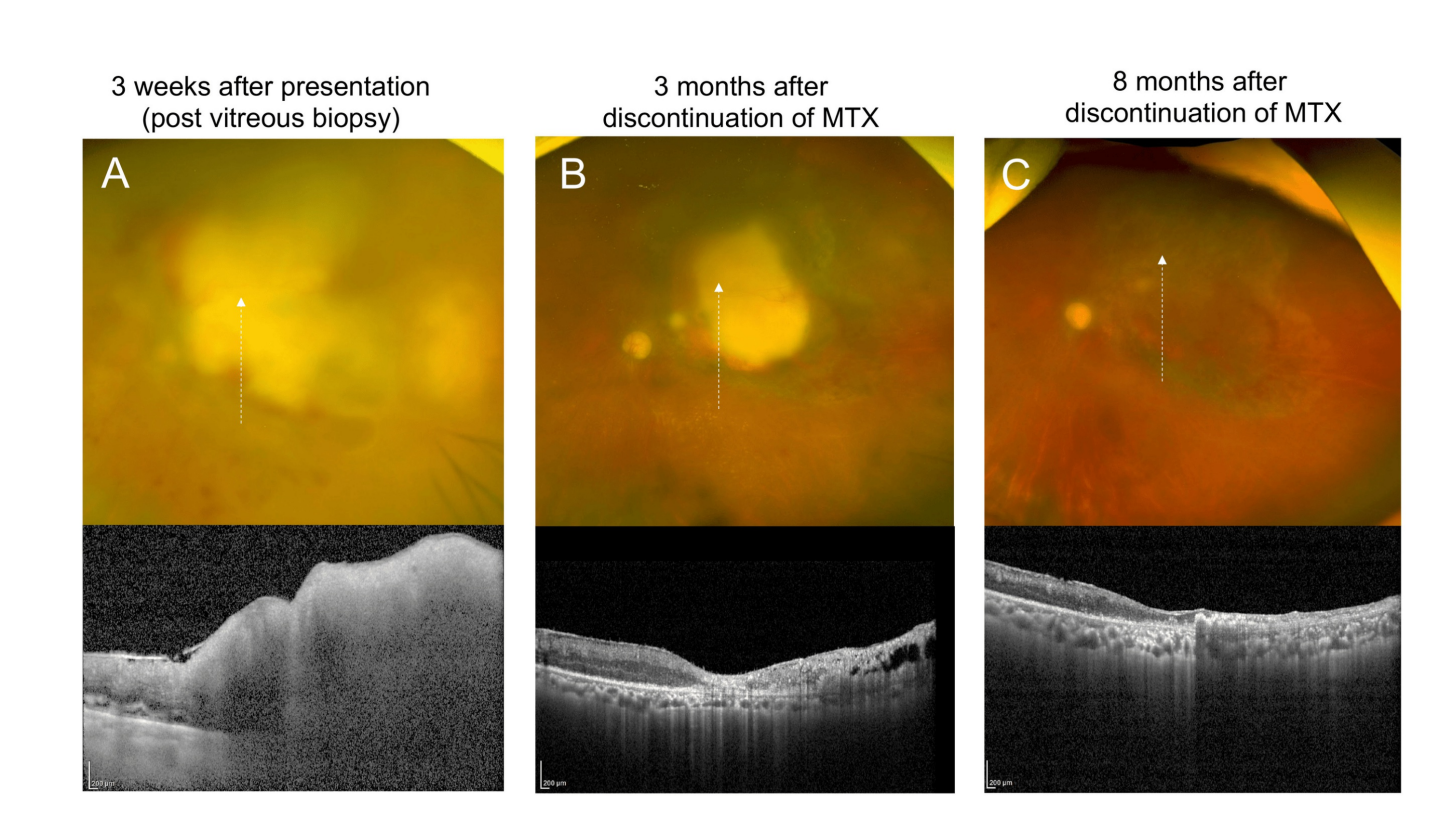
Fundus and OCT images in the left eye before and after discontinuation of MTX. (A) Large yellowish retinal mass on ultra-widefield fundus image (upper panel) and large inner retinal infiltrate on SD-OCT scan corresponding to yellowish retinal mass (lower panel). (B) Regression of yellowish retinal mass on ultra-widefield fundus image (upper panel) and reduced inner retinal infiltrate on SD-OCT scan at three months after MTX discontinuation (lower panel). (C) Resolution of yellowish retinal mass on ultra-widefield fundus image (upper panel) and retinal thinning on SD-OCT scan at eight months after discontinuation of MTX (lower panel). White dot arrow corresponds to scanned layer by OCT. OCT: optical coherence tomography, MTX: methotrexate.

Magnetic resonance imaging of the brain showed normal findings, and computed tomography showed right axillary lymph node enlargement. Although the patient was referred to a hematologist regarding biopsy of the enlarged axillary lymph node, the biopsy was not conducted due to her advanced age. Multiplex polymerase chain reaction (PCR) testing and quantitative PCR testing using an aqueous humor sample from the left eye revealed positive results for cytomegalovirus (CMV) (5.9 × 10² copies/mL) and Epstein-Barr virus (EBV) (2.7 × 10³ copies/mL). Since intraocular inflammation in both the anterior and posterior segments of the left eye was presumed to be related to CMV retinitis or MTX-LPD, topical corticosteroid eye drops (0.1% betamethasone) and intravitreal injection of ganciclovir (800 micrograms/0.1 mL) were initiated. Although discontinuation of MTX was proposed, the patient declined due to concerns about exacerbation of rheumatoid arthritis after MTX withdrawal. As visualization of the fundus in the left eye decreased due to progression of vitreous opacities two weeks after presentation, pars plana vitrectomy with vitreous biopsy was performed. Cytologic examination of the vitreous specimen showed class II findings. The interleukin (IL)-6 level was 11500 pg/mL, and the IL-10 level was below the detection limit in the vitreous sample. Analysis of immunoglobulin heavy-chain gene rearrangement was not performed due to insufficient sample quantity. Multiplex PCR testing and quantitative PCR testing using a vitreous sample disclosed positive results for CMV (1.8 × 10⁴ copies/mL) and EBV (1.1 × 10⁶ copies/mL). Although these findings provided inadequate evidence to support vitreoretinal lymphoma [6], the retinal mass lesion in the posterior pole grew rapidly. We explained the possible relationship between the ocular lesion and long-term MTX use for rheumatoid arthritis and obtained informed consent to discontinue MTX. MTX was withdrawn one month after presentation while treatment with abatacept was continued. Fundus examination and optical coherence tomography (OCT) showed gradual regression of the retinal mass in the posterior pole three months after MTX withdrawal (Figure 2A,B). The retinal mass lesion in the posterior segment nearly resolved eight months after MTX withdrawal (Figure 2C).

BCVA was no light perception in the left eye due to optic disc atrophy at eight months after discontinuation of MTX. Currently, the patient has been treated with abatacept and has maintained remission of rheumatoid arthritis, and no recurrence of the retinal mass has been observed during 13 months after withdrawal of MTX.

## Discussion

Extranodal lesions of MTX-LPD are observed in 40%-50% of patients with MTX-LPD, most commonly in the gastrointestinal tract, skin, liver, and lungs [4]. MTX-LPD rarely occurs in intraocular tissues [5]. Sone et al. reported the first case of MTX-LPD in intraocular tissues [5]. The patient was an 81-year-old Japanese woman with rheumatoid arthritis who had been treated with MTX (6 mg/week) for 15 years. Fundus examination revealed vitreous opacification in both eyes at presentation, and a diagnosis of intraocular MTX-LPD was made based on the results of cytology, IgH rearrangement, and the IL-10/IL-6 ratio using vitreous samples. The authors reported no detection of EBV DNA copies in vitreous or serum samples using real-time PCR, and no recurrence of vitreous opacity was observed during 13 months after cessation of MTX [5]. In the present study, although the results of cytology, IgH rearrangement, and the IL-10/IL-6 ratio using vitreous samples did not meet the criteria for vitreoretinal lymphoma as defined by Carbonell et al. [6], a diagnosis of MTX-LPD was made based on the medical history and clinical course showing regression of the retinal mass after discontinuation of MTX.

Tokuhira and colleagues demonstrated that 70% of patients with MTX-LPD showed EBV positivity [3]. The present case represents the first report of MTX-LPD demonstrating EBV positivity in both aqueous humor and vitreous fluid. In addition, the EBV copy number was high (1.1 × 10⁶ copies/mL) in the vitreous fluid. Several studies have reported that EBV positivity is significantly associated with spontaneous regression of MTX-LPD [7,8]. Yamamoto et al. demonstrated that EBV DNA was detected by qualitative PCR using ocular fluids in 28% of patients with uveitis, including infectious and non-infectious uveitis [9]. Since EBV can be nonspecifically detected in aqueous humor in cases of anterior uveitis by qualitative PCR testing, quantitative PCR analysis of EBV should be performed to help predict the clinical course after withdrawal of MTX in patients with MTX-LPD.

In the present case, quantitative PCR testing revealed positive results for CMV (5.9 × 10² copies/mL) and EBV (2.7 × 10³ copies/mL) in aqueous humor, and PCR testing using vitreous samples also disclosed positive results for CMV (1.8 × 10⁴ copies/mL) and EBV (1.1 × 10⁶ copies/mL). In this patient, treatment with abatacept, a biologic fusion protein that inhibits T-cell activation, along with long-term MTX use, could have led to an immunocompromised state, resulting in intraocular CMV infection. Indeed, Xia et al. reported a case of rheumatoid arthritis with development of CMV chronic retinal necrosis during treatment with abatacept and MTX [10]. Furthermore, a recent study reported a case without immunocompromise that was diagnosed as EBV-positive vitreoretinal lymphoma nearly one year after the diagnosis of CMV-associated anterior uveitis [11]. The authors proposed the hypothesis that chronic CMV infection facilitated the emergence of a clonal EBV-transformed B-cell population [11]. Further study is required to determine whether coinfection with CMV/EBV increases the risk of developing MTX-LPD in intraocular tissue.

## Conclusions

In the present study, a patient with a history of long-term MTX therapy for rheumatoid arthritis presented with EBV- and CMV-positive panuveitis and a retinal mass in the posterior pole. After cessation of MTX, the retinal mass gradually regressed. The present case indicates that intraocular MTX-LPD should be considered in the differential diagnosis of patients presenting with uveitis who are undertaking long-term MTX therapy. Multiplex PCR along with quantitative PCR for intraocular fluids may be helpful for prediction of the clinical course after stopping MTX and for understanding the pathogenesis of intraocular MTX-LPD.
